# Adjuvant Whole Breast Radiotherapy Improve Survival in Women with Heart Failure with Reduced Ejection Fraction Receiving Breast-Conserving Surgery

**DOI:** 10.3390/jpm11121358

**Published:** 2021-12-13

**Authors:** Jiaqiang Zhang, Shao-Yin Sum, Jeng-Guan Hsu, Ming-Feng Chiang, Tian-Shyug Lee, Szu-Yuan Wu

**Affiliations:** 1 Department of Anesthesiology and Perioperative Medicine, Henan Provincial People’s Hospital, People’s Hospital of Zhengzhou University, Zhengzhou 450052, China; jiaqiang197628@163.com; 2 Department of General Surgery, Lo-Hsu Medical Foundation, Lotung Poh-Ai Hospital, Yilan 265, Taiwan; b91401126@ntu.edu.tw; 3 Graduate Institute of Business Administration, Fu Jen Catholic University, New Taipei City 242062, Taiwan; peterjghsu@gmail.com (J.-G.H.); 036665@mail.fju.edu.tw (T.-S.L.); 4 Division of Gastroenterology and Hepatology, Department of Internal Medicine, Lo-Hsu Medical Foundation, Lotung Poh-Ai Hospital, Yilan 265, Taiwan; chiangmingf@gmail.com; 5 Department of Food Nutrition and Health Biotechnology, College of Medical and Health Science, Asia University, Taichung 413, Taiwan; 6 Big Data, Cancer Center, Lo-Hsu Medical Foundation, Lotung Poh-Ai Hospital, Yilan 265, Taiwan; 7 Division of Radiation Oncology, Lo-Hsu Medical Foundation, Lotung Poh-Ai Hospital, Yilan 265, Taiwan; 8 Department of Healthcare Administration, College of Medical and Health Science, Asia University, Taichung 413, Taiwan; 9 Centers for Regional Anesthesia and Pain Medicine, Taipei Municipal Wan Fang Hospital, Taipei Medical University, Taipei 110, Taiwan

**Keywords:** breast cancer, radiotherapy-related cardiotoxicity, breast-conserving surgery, radiotherapy, survival

## Abstract

Background: to date, no data on the effect of adjuvant whole breast radiotherapy (WBRT) on oncologic outcomes, such as all-cause death, locoregional recurrence (LRR), and distant metastasis (DM), are available in women with left-side breast invasive ductal carcinoma (IDC) and heart failure with reduced ejection fraction (HFrEF). Patients and Methods: we included 294 women with left-breast IDC at clinical stages IA–IIIC and HFrEF receiving breast-conserving surgery (BCS) followed by adjuvant WBRT or non-adjuvant WBRT. We categorized them into two groups based on their adjuvant WBRT status and compared their overall survival (OS), LRR, and DM outcomes. We calculated the propensity score and applied inverse probability of treatment weighting (IPTW) to create a pseudo-study cohort. Furthermore, we performed a multivariate analysis of the propensity score–weighted population to obtain hazard ratios (HRs). Results: in the IPTW-adjusted model, adjuvant WBRT (adjusted HR [aHR]: 0.60; 95% confidence interval [CI]: 0.44–0.94) was a significant independent prognostic factor for all-cause death (*p* = 0.0424), and the aHR (95% CI) of LRR and DM for adjuvant WBRT was 0.33 (0.24–0.71; *p* = 0.0017) and 0.37 (0.22–0.63; *p* = 0.0004), respectively, compared with the non-adjuvant WBRT group. Conclusion: Adjuvant WBRT was associated with a decrease in all-cause death, LRR, and DM in women with left IDC and HFrEF compared with non-adjuvant WBRT.

## 1. Introduction

Cardiovascular disease may be a complication of breast radiotherapy (RT) and the use of specific systemic agents in the treatment of breast cancer [1]. Incidental radiation to the heart as part of the initial treatment for breast cancer can result in a range of cardiotoxic effects, including coronary artery disease, cardiomyopathy, pericardial disease, valvular dysfunction, and conduction abnormalities [2,3,4]. At present, no recommended minimum radiation dose that is completely safe exists [3]. RT-related cardiotoxicity (RICT) is associated with a portion of the heart being placed in a radiation field [1]. For all patients with left-sided breast cancers, careful treatment planning is critical to minimize cardiac exposure to radiation [1]. 

The association of RT with cardiotoxicity is not dependent on the presence or absence of a breast but on the volume of radiation to the heart [3,4]. Thus, cardiotoxicities associated with RT differ between the postlumpectomy and postmastectomy settings; this is because in the postmastectomy setting, the RT field often includes the nodal tissues, and these nodes are not always targeted in the postlumpectomy setting [5,6]. Thus, postmastectomy RT is more often associated with cardiac disease relative to postlumpectomy RT, but this is likely a result of the usually larger irradiated volumes of the heart in postmastectomy RT [5,6]. Therefore, RICT in patients with breast cancer should be minimized by using different surgical techniques of breast-conserving surgery (BCS) and total mastectomy (TM). 

Another crucial issue is whether adjuvant whole breast RT (WBRT) can be safely given to women with heart failure (HF) and left-side breast cancer who receive BCS. No data are available to address the value of adjuvant WBRT in women with breast cancer and HF receiving BCS. HF due to left ventricle (LV) dysfunction is categorized according to LV ejection fraction (LVEF) as HF with reduced ejection fraction (LVEF ≤40%, known as HFrEF) [7,8,9]. Until now, no study has estimated the oncologic outcomes of adjuvant WBRT in women with breast-invasive ductal carcinoma (IDC) and HFrEF receiving BCS. 

## 2. Patients and Methods

### 2.1. Study Population

In this cohort study, data were retrieved from the Taiwan Cancer Registry Database (TCRD). Our study is the retrospective cohort study with propensity scores matching design. We included women with HF with reduced ejection fraction (LVEF ≤ 40%; HFrEF) [7,8,9] who had received a diagnosis of left-side breast IDC between 1 January 2008, and 31 December 2018. The index date was the date of BCS, and the follow-up duration was from the index date to 31 December 2019. The TCRD of the Collaboration Center of Health Information Application contains detailed cancer-related information of patients, including their clinical stage, pathologic stages, chemotherapy regimens, dose of chemotherapy, molecular status, drug use, hormone receptor status, radiation modalities and doses, and surgical procedures [10,11,12,13]. The study protocols were reviewed and approved by the Institutional Review Board of Tzu-Chi Medical Foundation (IRB109-015-B).

### 2.2. Inclusion and Exclusion Criteria

The diagnoses of the included patients with HFrEF were confirmed after their pathological data were reviewed, and the women with newly diagnosed left-side IDC were confirmed to have no other cancers or distant metastases. The women with HFrEF were included if they had received a left-side IDC diagnosis, were 20 years old or older, and had clinical stage IA–IIIC (American Joint Committee on Cancer [AJCC], 8th edition) without metastasis. Patients with HFrEF were excluded if they had a history of cancer before the IDC diagnosis date, unknown pathologic types, missing sex data, unclear staging, or non-IDC histology. In addition, patients undergoing neoadjuvant chemotherapy or with unclear differentiation of tumor grade, missing HR status, missing data on trastuzumab or anthracycline use, or unclear staging were excluded. Other adjuvant treatments such as adjuvant chemotherapy, hormone therapy, or the human epidermal growth factor receptor 2 inhibitors did not constitute exclusion criteria based on the National Comprehensive Cancer Network (NCCN) guidelines [14]. We also excluded patients with HFrEF with unclear data on surgical procedures such as BCS or TM, ill-defined nodal surgery, unclear Charlson comorbidity index (CCI), or unclear differentiation from our cohort. Hormone receptor positivity was defined as ≥1% of tumor cells demonstrating positive nuclear staining through immunohistochemistry [15]. All included women with breast cancer had no cardiac surgery. Therefore, we made sure that patients with severe valvular heart disease, severe three vessel disease, or left main coronary artery disease requiring cardiac intervention like percutaneous coronary intervention, coronary artery bypass grafting, valve replacement, or percutaneous balloon valvuloplasty were not included in the analysis.

After applying the inclusion and exclusion criteria, we included 294 women with HFrEF and AJCC clinical stage IA–IIIC and left-side IDC who had received a BCS followed by sentinel lymph node biopsy (SLNB) or axillary lymph node dissection (ALND) and divided them into two groups based on their adjuvant WBRT status to compare all-cause mortality: Group 1 (women with left-side IDC and HFrEF who received BCS followed by adjuvant WBRT) and Group 2 (women with left-side IDC and HFrEF who received BCS and non-adjuvant WBRT). We also excluded women in Group 1 receiving non-standard adjuvant WBRT (contrast with standard adjuvant radiotherapy consisting of irradiation to the whole breast with a minimum of 50 Gy). Contemporary RT techniques were included in our study and the conventional two-dimensional RT technique was excluded. The contemporary RT techniques included were three-dimensional RT and intensity-modulated radiation therapy. The incidence of comorbidities was scored using the CCI [16,17]. Coronary arterial diseases (one vessels stenosis or two vessels stenosis), valvular disease (aortic stenosis/regurgitation or mitral stenosis/regurgitation), hypertension, atrial fibrillation, cardiomyopathy, ventricular arrhythmias, and diabetes were excluded from the CCI scores to avoid repetitive adjustment in multivariate analysis. The definition of cardiomyopathy in our study was idiopathic dilated cardiomyopathy. Only comorbidities observed within 6 months before the index date were included; they were coded and classified according to the International Classification of Diseases, 10th Revision, Clinical Modification (ICD-10-CM) codes at the first admission or based on more than two repetitions of a code issued at outpatient department visits.

### 2.3. Study Covariates and Statistical Analysis

Significant independent predictors, namely age, diagnosis year, CCI score, differentiation, pT, pN, hypertension, hyperlipidemia, CKD, coronary arterial diseases (one-vessels stenosis or two-vessel stenosis), valvular disease (aortic stenosis/regurgitation or mitral stenosis/regurgitation), atrial fibrillation, cardiomyopathy, ventricular arrhythmias, diabetes, statins, antithrombotic therapy, diuretic, beta blockers, and renin-angiotensin system inhibitor, chemotherapy with anthracycline-based regimen, hormone receptor status, trastuzumab use, nodal surgery, hospital level (academic or nonacademic), and income (Table 1), were analyzed using a multivariate analysis of the propensity score–weighted population to determine hazard ratios (HRs). We calculated the propensity score and applied inverse probability of treatment weighting (IPTW) to create a pseudo-study cohort; the weighted cohort avoids covariate bias and mimics randomized adjuvant WBRT or non-adjuvant WBRT assignment: IPTW for patients with WBRT = 1/p(WBRT); IPTW for patients without WBRT = 1/(1 − p[WBRT]) [18,19]. The independent predictors were examined in multivariable analyses after IPTW adjustment. Moreover, they were controlled for and were stratified in the analysis. The endpoint was all-cause death in the women with left-side IDC and HFrEF who received BCS followed by adjuvant WBRT (Group 1, case group) and in the women with left IDC and HFrEF who received BCS and had non-adjuvant WBRT (Group 2, control group). 

The cumulative incidence of death was estimated using the Kaplan–Meier method, and differences in the overall survival (OS), locoregional recurrence (LRR)-free survival, and distant metastasis (DM)-free survival between women with left IDC and HFrEF receiving BCS followed by adjuvant WBRT versus non-adjuvant WBRT were determined using a log-rank test. After confounders were adjusted for, IPTW-adjusted models were used to determine the time from the index date to all-cause mortality in the women with left IDC and HFrEF who received BCS followed by adjuvant WBRT or non-adjuvant WBRT. Subsequently, in a multivariate analysis, HRs were adjusted for covariates mentioned in Table 2. All analyses were conducted using SAS (Version 9.4; SAS, Cary, NC, USA), and a two-tailed *p* value < 0.05 was considered statistically significant.

## 3. Results

### 3.1. Study Cohort

We included 294 women with left-breast IDC at clinical stages IA–IIIC and HFrEF who received BCS followed by adjuvant WBRT or non-adjuvant WBRT (Table 1). Among these women, 223 with left IDC and HFrEF received BCS followed by adjuvant WBRT (Group 1) and 71 with left IDC and HFrEF received BCS with non-adjuvant WBRT (Group 2). After IPTW was executed using the propensity score, the covariates between Groups 1 and 2 were found to be homogenous. The median follow-up durations after the index date were 6.96 and 5.09 years for women with left IDC and HFrEF who received BCS followed by adjuvant WBRT or non-adjuvant WBRT, respectively. All standardized differences in covariates were smaller than 0.1 (Table 1) and were homogenous between the two groups. The prevalence of hyperlipidemia were 43.9% and 47.9% for adjuvant WBRT and no-adjuvant WBRT groups, respectively.

### 3.2. Effects of Adjuvant Whole Breast Radiotherapy (WBRT) on Oncologic Outcomes in Women with Left-Side Invasive Ductal Carcinoma (IDC) and Heart Failure with Reduced Ejection Fraction (HFrEF) Receiving Breast-Conserving Surgery (BCS) 

IPTW-adjusted models indicated that adjuvant WBRT was a significantly better independent prognostic factor for OS, LRR, and DM in the women with left IDC and HFrEF receiving BCS (Table 2). Adjuvant WBRT (adjusted HR [aHR]: 0.60; 95% confidence interval [CI]: 0.44–0.94) was a significant independent prognostic factor for all-cause death (*p* = 0.0424; Table 2). In the IPTW-adjusted model, the aHR (95% CI) for LRR in the adjuvant WBRT group was 0.33 (0.24–0.71; *p* = 0.0017; Table 2) compared with the non-adjuvant WBRT group. Moreover, the aHR (95% CIs) for DM in the adjuvant WBRT group was 0.37 (0.22–0.63; *p* = 0.0004) compared with the non-adjuvant WBRT group (Table 2). 

### 3.3. Other Independent Predictors of All-Cause Death, Locoregional Recurrence (LRR), and Distant Metastasis (DM) in the Women with Left IDC and HFrEF Receiving BCS

Old age (>65 years), CCI ≥ 1, advanced pT stages (pT2–4), advanced pN stages (pN1–3), hormone receptor negative status, and differentiation Grade II and III were identified as crucial independent poor prognostic factors for OS (Table 2). IPTW-adjusted models were adjusted for age, diagnosis year, CCI score, differentiation, pT, pN, hypertension, ischemic heart disease, heart valvular disease, cardiomyopathy, arrhythmias and conduction disorders, diabetes, adjuvant chemotherapy with anthracycline-based regimen, hormone receptor status, trastuzumab use, nodal surgery, hospital level, and income; the aHRs (95% CIs) of all-cause death for age > 65 years, CCI ≥ 1, pT2–4, pN1–3, differentiation Grades II and III, and hormone receptor positive status were 1.31 (1.12–3.01), 1.15 (1.09–1.66), 1.28 (1.05–2.28), 2.31 (1.30–4.24), 1.44 (1.10–2.00), 1.71 (1.24–2.13), and 0.70 (0.50–0.89) compared with age 20–65 years, CCI = 0, pT1, pN0, differentiation grade I, and hormone receptor negative status, respectively (Table 2). IPTW-adjusted models also revealed the aHRs (95% CIs) of LRR for pT2–4, pN1–3, differentiation grade II, differentiation grade III, and hormone receptor positive status to be 1.69 (1.03–2.73), 1.72 (1.35–4.30), 1.86 (1.43–2.18), 1.91 (1.44–2.56), and 0.68 (0.51–0.88) compared with pT1, pN0, differentiation grade I, and hormone receptor negative status, respectively. Moreover, the aHRs (95% CIs) of DM for pT2–4, pN1-3, differentiation grade II, differentiation grade III, and hormone receptor positive status were 1.41 (1.18–3.17), 1.66 (1.24–2.69), 1.82 (1.39–2.79), 1.96(1.51–2.67), and 0.61 (0.38–0.74) compared with pT1, pN0, differentiation grade I, and hormone receptor negative status, respectively.

### 3.4. Survival Curves of Adjuvant WBRT or Non-Adjuvant WBRT in Women with Left IDC and HFrEF Receiving BCS 

**Figure 1:**
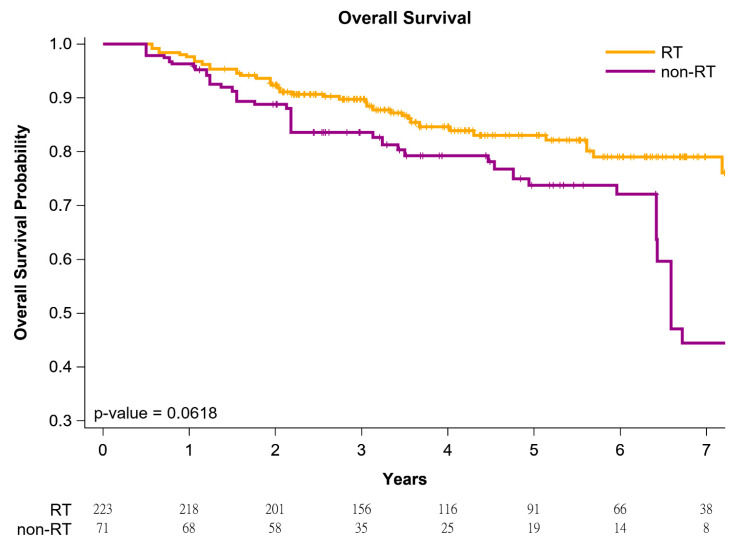
Kaplan–Meier overall survival curves of propensity score–weighted population with breast cancer and heart failure with reduced ejection fraction receiving breast conservative surgery.

**Figure 2:**
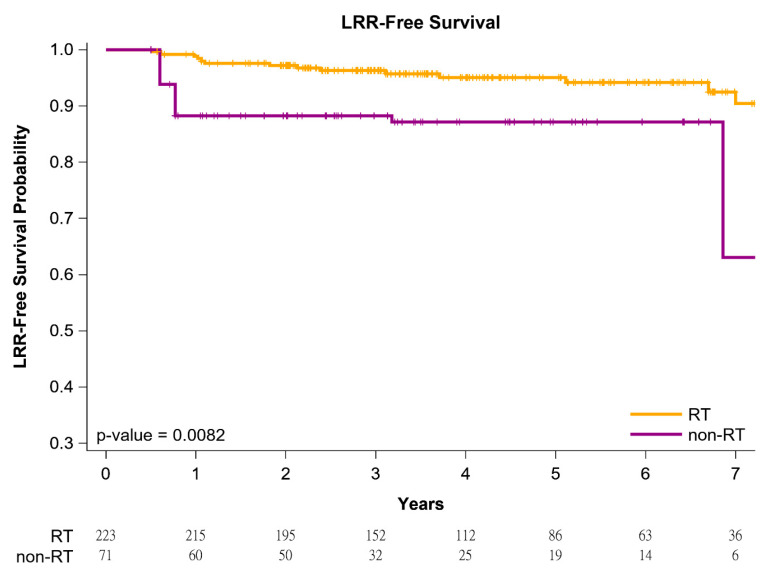
Kaplan–Meier locoregional recurrence-free survival curves of propensity score–weighted population with breast cancer and heart failure with reduced ejection fraction receiving breast conservative surgery.

**Figure 3:**
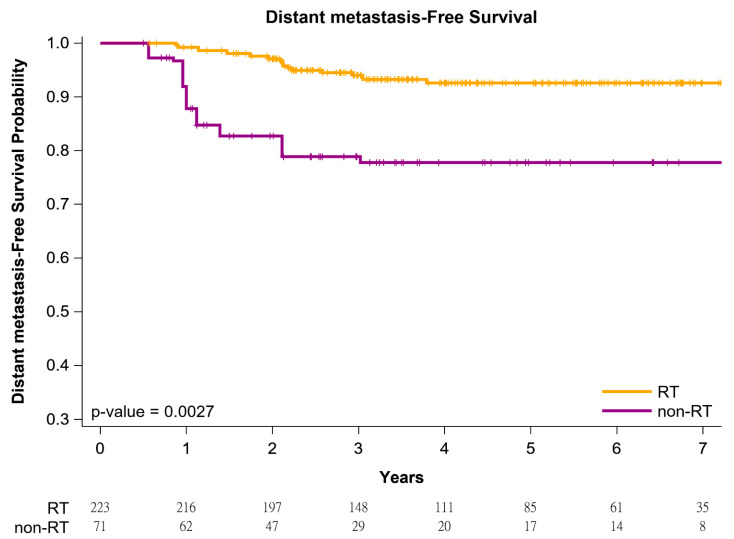
Kaplan–Meier distant metastasis–free survival curves of propensity score–weighted population with breast cancer and heart failure with reduced ejection fraction receiving breast conservative surgery.

Figure 1 presents Kaplan–Meier curves that illustrate the OS of the women with left IDC and HFrEF receiving BCS with adjuvant WBRT or non-adjuvant WBRT. The 5-year overall survival rates were 86.47% and 75.92% in the adjuvant WBRT and non-adjuvant WBRT groups, respectively (Figure 1); the OS rate was associated with an increasing trend in the adjuvant WBRT group (log-rank test, *p* = 0.0618) compared with the non-WBRT group. Additionally, the 5-year LRR-free survival in women with left IDC and HFrEF receiving BCS was 95.78% and 86.11% in the adjuvant WBRT group and non-adjuvant WBRT group, respectively (Figure 2; log-rank test, *p* = 0.0083). The 5-year DM-free survival in women with left IDC and HFrEF receiving BCS was 96.23% and 78.33% in the adjuvant WBRT group and non-adjuvant WBRT group, respectively (Figure 3; log-rank test, *p* = 0.0027).

## 4. Discussion

The use of RT has contributed to significant improvements in disease-specific survival among patients with early stage breast cancer [20]. The success of RT, used either alone or in combination with other modalities, has resulted in large cohorts of breast cancer survivors who are vulnerable to late complications such as RICT from RT [5,21,22,23,24,25,26,27]. Numerous treatment-related factors are responsible for cardiotoxicity in women with breast cancer [28,29,30,31,32,33,34,35,36,37,38]. Thus, we conducted the study to determine the survival benefits offered by adjuvant WBRT in women with left-side IDC and HFrEF receiving BCS.

Patients with breast cancer might experience adverse effects from many cardiotoxic treatments such as adjuvant RT, anthracycline-based chemotherapy, or trastuzumab [5,21,22,23,24,25,26,27,28,29,30,31,32,33,34,35,36,37,38]. Although cardiovascular diseases such as HF, heart attacks, and stroke remain the leading causes of death in women, many believe breast cancer to be more deadly [39]. In fact, the risk of RICT should be weighed against the potential benefits of adjuvant WBRT with respect to the patients’ prognosis and likely clinical benefit [5,21,22,23,24,25,26,27]. Until now, no data have been available for the evaluation of oncologic outcomes (OS, LRR, and DM) of adjuvant WBRT in women with left-side breast IDC and HFrEF receiving BCS. This is the first study to explore the value of adjuvant WBRT for women with left-side breast IDC and HFrEF receiving BCS. As shown in Table 2, adjuvant WBRT resulted in better OS, LRR-free status, and DM-free status compared with non-adjuvant WBRT in women with left-side breast IDC and HFrEF receiving BCS. The potential reasons might be the recent decline in mortality in women with HF [40,41] and the advances in contemporary RT techniques with reduced irradiation volumes to the heart [2,23,24].

According to our literature review, this is the first study to estimate the oncologic outcomes of adjuvant WBRT among women with left-side breast IDC and HFrEF receiving BCS. There is no consensus or evidence for the use of adjuvant WBRT in women with left-side breast IDC and HFrEF receiving BCS. In the IPTW-adjusted models, adjuvant WBRT was associated with a decrease in the risk of all-cause death, LRR, and DM among women with left-side breast IDC and HFrEF receiving BCS (Table 2). We have no ethical conflicts in the study. We only presented the real world data compatible with our daily practice experience in Taiwan. In fact, some physicians choose BCS only and non-adjuvant left-side breast RT for women with breast cancer having HFrEF in the real world. The potential reasons might be the concerns that left-side breast radiotherapy might aggravate heart failure contributing to cardiac death before breast cancer death. Our study is the first to demonstrate that the potential benefits of adjuvant WBRT with contemporary RT techniques outweighs the risk of RICT given the patients’ prognosis and likely long-term OS, LRR, and DM benefits (Table 2). According to our findings, we strongly suggest that women with left-side breast IDC and HFrEF receiving BCS should also receive adjuvant WBRT to decrease the risk of all-cause death, LRR, and DM.

A strength of our study was that it was the first cohort study to estimate the survival outcomes of adjuvant WBRT or non-adjuvant WBRT among women with left-side IDC and HFrEF receiving BCS. The covariates between the adjuvant WBRT and non-adjuvant WBRT groups were homogenous for women with left-side IDC and HFrEF receiving BCS, with no selection bias (Table 1). No study has estimated the effect of adjuvant WBRT on women with left-side IDC and HFrEF receiving BCS. In our study, the poor prognostic factors for OS in women with left-side IDC and HFrEF receiving BCS were old age, CCI ≥1, advanced pT stages (pT2–4), advanced pN stages (pN1–3), hormone receptor negative status, and differentiation Grades II–III of (Table 2), which are consistent with factors in women with breast cancer without HFrEF reported in previous studies [42,43,44,45,46]. Furthermore, our study is the first to demonstrate the benefits of adjuvant WBRT with contemporary RT techniques for OS, LRR, and DM in women with left-side IDC and HFrEF receiving BCS. Our findings should be considered in future clinical practice and prospective clinical trials. We suggest that adjuvant WBRT is valuable to achieving better outcomes of OS, LRR, and DM in women with left-side IDC and HFrEF receiving BCS.

This study has some limitations. First, because all women with left-side breast IDC and HFrEF were included from an Asian population, the corresponding ethnic susceptibility compared with the non-Asian population remains unclear; hence, our results should be cautiously extrapolated to non-Asian populations. However, no evidence exists as to the differences in oncologic outcomes in Asian versus non-Asian patients with breast IDC and HFrEF receiving BCS. Second, the factors of New York Heart Association functional class [47], magnitude of left ventricular ejection fraction, left ventricular hypertrophy, left atrial dilatation, brain natriuretic peptide levels cannot be available in the TCRD. However, all coding of HFrEF was determined by professional cardiologists in Taiwan and the coding was all by craniological specialists. The cardiovascular diseases were verified as accurate by the previous cardiovascular studies. Therefore, we believe the definition of HFrEF will be specific and accurate. Third, the diagnoses of all comorbid conditions were based on ICD-10-CM codes. However, the combination of Taiwanese TCRD and National Health Insurance Research Database (NHIRD) data appears to be a valid resource for population research on cardiovascular diseases, stroke, or chronic comorbidities [48,49,50]. Moreover, the Taiwan Cancer Registry Administration randomly reviews charts and interviews patients to verify the accuracy of the diagnoses, and hospitals with outlier chargers or practices may be audited and subsequently be heavily penalized if any malpractice or discrepancy is detected. Accordingly, to obtain crucial information on population specificity and disease occurrence, a large-scale randomized trial comparing carefully selected patients undergoing suitable treatments is essential. Finally, the TCRD does not contain information regarding dietary habits or body mass index, which may be risk factors for mortality. Nevertheless, considering the magnitude and statistical significance of the observed effects in this study, these limitations are unlikely to affect the conclusions.

## 5. Conclusions

Adjuvant WBRT was associated with a decrease in all-cause death, LRR, and DM among women with left-side breast IDC and HFrEF compared with non-adjuvant WBRT. We suggest adjuvant WBRT for women with left-side IDC receiving BCS, even if they have HFrEF.

## Figures and Tables

**Table 1:** Demographics of patients with breast cancer and heart failure with reduced ejection fraction who received breast conservative surgery in the propensity score–weighted population through inverse probability of treatment weighting (IPTW).

		Propensity Score–Weighted Population				
		Adjuvant WBRT N = 223		Non-Adjuvant WBRT N = 71		
Variable		*n*	(%)	*n*	(%)	Standardized Difference
Age	Mean (SD)	62.3	(11.4)	63.5	(11.6)	0.0311
	Median (IQR, Q1–Q3)	63	(54–70)	63	(54–71)	
	20–65	167	(73.6)	52	(73.2)	0.0224
	>65	60	(26.4)	19	(26.8)	
Diagnosis year	2008–2012	89	(39.2)	28	(39.4)	0.0000
	2013–2017	138	(60.8)	43	(60.6)	
CCI score	0	73	(32.7)	22	(31.0)	0.0231
	≥1	150	(67.3)	49	(69.0)	
Differentiation	I	44	(19.7)	16	(22.5)	-
	II	112	(50.2)	34	(47.9)	0.0566
	III	67	(30.0)	21	(29.6)	0.0452
AJCC clinical stage	I	112	(50.2)	33	(46.5)	0.0831
	II–III	111	(49.8)	38	(53.5)	
AJCC pathologic stage	I	109	(48.9)	34	(47.9)	-
	II	97	(43.5)	32	(45.1)	0.0242
	III	18	(8.1)	6	(8.5)	0.0170
pT	pT1	137	(61.4)	41	(57.7)	0.0737
	pT2–4	86	(38.6)	30	(42.3)	
pN	pN0	164	(73.5)	49	(69.0)	0.0705
	pN1–3	59	(26.5)	22	(31.0)	
Hypertension		161	(72.2)	50	(70.4)	0.0584
Hyperlipidemia		98	(43.9)	34	(47.9)	0.0871
CKD		67	(30.0)	32	(45.1)	0.0171
Coronary arterial diseases		88	(39.5)	27	(38.0)	0.0352
	One-vessel stenosis	60	(26.9)	18	(25.4)	
	Two-vessel stenosis	28	(12.6)	9	(12.7)	
Valvular disease		17	(7.6)	6	(8.5)	0.0276
	Aortic stenosis/regurgitation	10	(4.5)	3	(4.2)	
	Mitral stenosis/regurgitation	7	(3.1)	3	(4.2)	
Atrial fibrillation		60	26.9	19	26.8	0.0592
Cardiomyopathy		71	(31.8)	23	(32.4)	0.0381
Ventricular arrhythmias		6	(2.7)	2	(2.8)	0.0114
Diabetes mellitus		76	(34.1)	27	(38.0)	0.0779
Statins		58	(26.0)	20	(28.2)	0.0923
Antithrombotic therapy		78	(35.0)	28	(39.4)	0.0610
Diuretic		89	(39.9)	29	(40.8)	0.0276
Beta blockers		133	(59.6)	46	(64.8)	0.0110
Renin-angiotensin system inhibitor		166	(74.4)	51	(71.8)	0.0853
Chemotherapy with anthracycline		103	(46.2)	34	(47.9)	0.0072
Hormone receptor positive		109	(48.9)	35	(49.3)	0.0089
Trastuzumab use		21	(9.3)	7	(9.9)	0.0131
Nodal surgery	ALND	139	(61.2)	40	(56.3)	0.0618
	SLNB	84	(38.8)	31	(43.7)	
Hospital level	Academic center	110	(49.3)	35	(49.3)	0.0000
	Non-academic center	113	(50.7)	36	(50.7)	
Income	<NTD 18,000	65	(29.1)	20	(28.2)	-
	NTD 18,000–24,000	74	(33.2)	23	(32.4)	0.0231
	NTD 24,000–36,000	40	(17.9)	12	(16.9)	0.0143
	>NTD 36,000	44	(19.7)	16	(22.5)	0.0712

Abbreviations: IQR, interquartile range; SD, standard deviation; AJCC, American Joint Committee on Cancer; Her-2, Human Epidermal Growth Factor Receptor-2; WBRT, whole-breast radiotherapy; CCI, Charlson comorbidity index; T, tumor; N, nodal; pT, pathologic tumor stage; pN, pathologic nodal stage; ALND, axillary lymph node dissection; SNLB, sentinel lymph node biopsy; NTD, New Taiwan dollar.

**Table 2:** Multivariate analysis of propensity score–weighted population with breast cancer and heart failure with reduced ejection fraction receiving breast conservative surgery.

		All-Cause Death			Local Recurrence			Distant Metastasis		
		aHR *	(95% CI)	*p* Value	aHR *	(95% CI)	*p* Value	aHR *	(95% CI)	*p* Value
Adjuvant WBRT	No	Ref		0.0424	Ref		0.0017	Ref		0.0004
	Yes	0.60	(0.44–0.94)		0.33	(0.24–0.71)		0.37	(0.22–0.63)	
Age	20–65	Ref		0.0001	Ref		0.5641	Ref		0.5028
	>65	1.31	(1.12–3.01)		1.09	(0.71–1.74)		1.49	(0.72–1.79)	
Diagnosis year	2009–2012	Ref		0.4796.	Ref		0.2554	Ref		0.7472
	2013–2017	0.83	(0.76–1.38)		0.75	(0.53–1.11)		0.91	(0.63–1.58)	
CCI score	0	Ref		0.0385	Ref		0.5028	Ref		0.7631
	≥1	1.15	(1.09–1.66)		1.03	(0.70–1.68)		1.02	(0.57–1.55)	
Hypertension	Yes	1.15	(0.70–1.88)	0.5482	0.93	(0.61–1.43)	0.8204	0.95	(0.69–1.33)	0.8861
Hyperlipidemia	Yes	1.06	(0.66–2.01)	0.6431	0.94	(0.61–1.61)	0.8421	0.92	(0.65–1.61)	0.8141
CKD	Yes	1.17	(0.88–1.76)	0.5157	0.88	(0.70–1.81)	0.7315	0.82	(0.59–1.39)	0.6929
Coronary arterial diseases	No	Ref		0.3868	Ref		0.5628	Ref		0.5806
	One-vessel stenosis	1.26	(0.77–1.98)		0.81	(0.52–1.19)		0.88	(0.71–1.44)	
	Two-vessel stenosis	1.36	(0.80–2.33)		0.77	(0.50–1.23)		0.87	(0.78–1.57)	
Valvular disease	No	Ref		0.3598	Ref		0.6724.	Ref		0.6682
	Aortic stenosis/regurgitation	1.16	(0.76–2.18)		0.98	(0.61–1.41)		0.96	(0.54–1.77)	0.7963
	Mitral stenosis/regurgitation	1.17	(0.74–2.10)		0.94	(0.59–1.33)		0.95	(0.50–1.91)	0.8190
Atrial fibrillation	Yes	1.08	(0.76–1.40)	0.3200	0.79	(0.49–1.80)	0.4219	0.90	(0.67–2.50)	0.5893
Cardiomyopathy	Yes	1.13	(0.88–1.65)	0.1312	0.77	(0.45–1.82)	0.3404	0.92	(0.63–2.57)	0.5322
Ventricular arrhythmias	Yes	1.08	(0.88–1.46)	0.1786	0.89	(0.53–2.67)	0.2762	0.76	(0.41–1.39)	0.3174
Diabetes	Yes	1.06	(0.84–1.14)	0.3127	0.91	(0.67–1.20)	0.4714	0.68	(0.52–1.28)	0.8784
Statins	Yes	0.91	(0.70–1.65)	0.4387	0.94	(0.55–1.76)	0.5494	0.90	(0.54–1.91)	0.5499
Antithrombotic therapy	Yes	1.03	(0.77–1.51)	0.2609	0.97	(0.60–2.11)	0.4980	0.88	(0.51–1.90)	0.4251
Diuretic	Yes	1.01	(0.60–2.60)	0.4427	0.95	(0.58–2.29)	0.5068	0.89	(0.53–2.42)	0.5778
Beta blockers	Yes	0.88	(0.68–1.43)	0.2080	0.90	(0.50–2.99)	0.4050	0.86	(0.44–1.84)	0.3160
Renin-angiotensin system inhibitor	Yes	0.86	(0.54–1.97)	0.5810	0.96	(0.86–2.36)	0.4169	0.87	(0.57–1.81)	0.5308
pT	pT1	Ref		0.0217	Ref		0.0316	Ref		0.0245
	pT2–4	1.28	(1.05–2.28)		1.69	(1.03–2.73)		1.41	(1.18–3.17)	
pN	pN0	Ref		0.0017	Ref		0.0072	Ref		0.0153
	pN1–3	2.31	(1.30–4.24)		1.72	(1.35–4.30)		1.66	(1.24–2.69)	
Differentiation	I	Ref		0.0003	Ref		0.0001	Ref		0.0001
	II	1.44	(1.10–2.00)		1.86	(1.43–2.18)		1.82	(1.39–2.79)	
	III	1.71	(1.24–2.13)		1.91	(1.44–2.56)		1.96	(1.51–2.67)	
Chemotherapy with anthracycline	Yes	0.97	(0.35–1.54)	0.2653	0.80	(0.39–1.81)	0.2876	0.82	(0.60–1.77)	0.4876
Hormone receptor positive	Yes	0.70	(0.50–0.89)	0.0085	0.68	(0.51–0.88)	0.0028	0.61	(0.38–0.74)	0.0016
Trastuzumab use	Yes	1.05	(0.48–2.50)	0.8893	1.60	(0.71–3.70)	0.8974	1.08	(0.70–1.88)	0.6432
Nodal surgery	ALND	Ref		0.8742	Ref		0.3682	Ref		0.3531
	SLNB	1.12	(0.93–1.24)		1.25	(0.81–2.25)		1.37	(0.71–2.78)	
Hospital level	Medical centers	Ref		0.1667	Ref		0.4539	Ref		0.7830
	Non-medical centers	1.09	(0.80–1.25)		0.91	(0.60–2.55)		0.95	(0.70–1.50)	
Income	<NTD 18,000	Ref		0.4267	Ref		0.8541	Ref		0.7652
	NTD 18,000–24,000	1.31	(0.60–2.94)		1.21	(0.68–2.16)		1.14	(0.65–1.98)	
	NTD 24,000–36,000	1.51	(0.85–2.67)		1.38	(0.71–2.47)		1.51	(0.76–2.19)	
	>NTD 36,000	1.66	(0.90–3.00)		1.71	(0.92–2.98)		2.11	(0.81–3.22)	

Abbrevations: aHR, adjusted hazard ratios; CIs, confidence intervals; HR, hormone receptor; Her-2, human epidermal growth factor receptor-2; WBRT, whole-breast radiotherapy; CCI, Charlson comorbidity index; T, tumor; N, nodal; pT, pathologic tumor stage; pN, pathologic nodal stage; ALND, axillary lymph node dissection; SNLB, sentinel lymph node biopsy; ref, reference group; NTD, New Taiwan dollar. * All covariates mentioned in Table 2 were adjusted.

## Data Availability

Restrictions apply to the availability of these data. Data were obtained from the Health and Welfare Data Science Center and are available with the permission of the Health and Welfare Data Science Center, Taiwan.

## Abbreviations

WBRT	whole breast radiotherapy
LRR	locoregional recurrence
DM	distant metastasis
IDC	invasive ductal carcinoma
HFrEF	heart failure with reduced ejection fraction
BCS	breast-conserving surgery
OS	overall survival
aHR	adjusted hazard ratio
HR	hazard ratio
IPTW	inverse probability of treatment weighting
CI	confidence interval
AJCC	American Joint Committee on Cancer
TCRD	Taiwan Cancer Registry Database
SD	standard deviation
Her-2	human epidermal growth factor receptor-2
SLNB	sentinel lymph node biopsy
ALND	axillary lymph node dissection
CKD	chronic kidney disease
CCI	Charlson comorbidity index
ICD-10-CM	International Classification of Diseases, 10th Revision, Clinical Modification
NCCN	National Comprehensive Cancer Network
RT	radiotherapy
RICT	radiotherapy-related cardiotoxicity
TM	total mastectomy
HF	heart failure
LV	left ventricular
LVEF	left ventricular ejection fraction
T	tumor
N	nodal
pT	pathologic tumor stage
pN	pathologic nodal stage
NHIRD	National Health Insurance Research Database
